# Synergistic Interaction Between High Bioactive IL-17A and Joint Destruction for the Occurrence of Cardiovascular Events in Rheumatoid Arthritis

**DOI:** 10.3389/fimmu.2020.01998

**Published:** 2020-08-26

**Authors:** Marie Robert, Arnaud Hot, François Mifsud, Ndiémé Ndongo-Thiam, Pierre Miossec

**Affiliations:** ^1^Department of Clinical Immunology and Rheumatology, Immunogenomics and Inflammation Research Unit EA 4130, University of Lyon, Lyon, France; ^2^Department of Internal Medicine, Immunogenomics and Inflammation Research Unit EA 4130, University of Lyon, Lyon, France

**Keywords:** interleukin-17, rheumatoid arthritis, cardiovascular diseases, joint destruction, cardiovascular prevention

## Abstract

Rheumatoid arthritis (RA) remains a cause of morbidity and mortality in many patients while new treatments have changed the face of the disease. Despite the emergence of these new drugs, cardiovascular (CV) diseases remain more frequent in RA patients compared with the general population. However, predictive biomarkers of RA severity and precise guidelines to manage the CV risk in these patients are still lacking. Pro-inflammatory cytokines contribute both to RA and CV pathogenesis. Focusing on IL-17A, high levels of bioactive IL-17A were associated with destruction in RA but also during myocardial infarction. The study aimed to assess the relationship between bioactive IL-17A, destruction and the occurrence of CV events (CVE) in RA patients with a very long follow-up. Thirty-six RA patients were followed between 1970 and 2012 in Lyon, France. They were tested for bioactive IL-17A and clinical and biological characteristics were recorded at baseline. Then, the occurrence of CVE was registered during the follow-up. To study the bioactive fraction of IL-17A, the bioassay used the ability of human umbilical vein endothelial cells to produce IL-8 in presence of RA plasma samples with or without an anti-IL-17A antibody. Bioactive IL-17A level at baseline was higher in RA patients who later experienced a CVE compared to those without (0.77 vs 0.21 ng/ml, *p*-value = 0.0095, Mann-Whitney test) and synergized with joint destruction (*p*-value = 0.020, Kruskal-Wallis test). Through its effects on vessels and thrombosis, high levels of bioactive IL-17A could represent a long-term marker of CV risk.

## Introduction

Active rheumatoid Arthritis (RA) leads to joint destruction but is also associated with premature cardiovascular events (CVE) (1). Thirty percent of CVE in RA patients can be attributed to RA inflammation-related parameters (joint destruction, auto-antibodies), and, these parameters combined with traditional CV risk factors could account for up to 70% of all CVE (2). It remains crucial to identify predictive biomarkers of CVE in RA and to institute guidelines to manage this CV risk. These biomarkers could be pro-inflammatory cytokines as chronic inflammation contributes to both CV pathogenesis and RA (3). Among them, interleukin (IL)-17A plays a role both in RA development and progression, and increases cardiovascular risk by inducing inflammation, coagulation and thrombosis (4–7). Because the effect of circulating IL-17A can be positively or negatively modulated by other mediators [e.g., tumor necrosis factor-alpha (TNFα), IL-25], it is critical to focus on the bioactive fraction of IL-17A that takes into account these interactions (8). A bioassay was previously developed to study specifically bioactive IL-17A. Using this bioassay, increased levels of bioactive IL-17A were found in RA patients with joint destruction compared to those without (9) and patients with myocardial infarction showed a peak of bioactive IL-17A at admission (10).

To investigate the link between these two aspects and to identify predictive biomarkers of CVE in RA, this bioassay was used to measure bioactive IL-17A from 36 RA patients with a very long follow-up.

## Materials and Methods

### Patients

Patients included in this study were followed between 1970 and 2012 at the Clinical Immunology and Rheumatology department in a tertiary hospital in Lyon, France. Patients have to fulfill the American College of Rheumatology criteria for RA diagnosis (11). Clinical data were collected for each patient and recorded in a computer database from the beginning to the end of the follow-up. For the 36 RA patients included in this study, biological samples were collected at time of entry. Samples were then kept frozen in a biocollection. Patients with history of CVE before sample collection were excluded. Parameters were collected at the time of IL-17A sample collection: joint destruction, assessed using the wrist Larsen score, age, sex, anti-citrullinated protein antibodies (ACPA) positivity and disease activity score-28 (DAS-28). RA was considered as destructive if the wrist Larsen score was ≥2, as non-destructive if the Larsen score was equal to 0 or 1. Then, the occurrence of CVE, defined as myocardial infarction, stroke and peripheral acute ischemia, was recorded during the follow-up. Efforts were made so that at sample collection, patients with CVE were comparable with patients without CVE in terms of age, sex, ACPA positivity and DAS-28. All patients signed an informed consent. The study complied with the local Ethical Committee of the Hospitals of Lyon and was approved by the Ministry of Research (reference number: AC-2010-1164).

### Functional Assay for Bioactive IL-17A

To explore the link between the occurrence of CVE, joint destruction and the circulating IL-17 bioactivity, a functional assay was performed (9). Blood samples from 36 RA patients were collected and kept frozen in a bio-collection. The assay is based on the ability of human umbilical vein endothelial cells (HUVEC) to produce IL-8 in response to inflammatory cytokines (e.g., TNFα, IL-25) that are present in RA plasma samples. To measure the specific contribution of IL-17A in IL-8 production, plasma samples (diluted at 10%) are first pre-incubated with or without a blocking anti-IL-17A monoclonal antibody (10 μg/ml, R&D Systems, Paris, France) and then added to HUVEC for 48 h. The difference between IL-8 production by ELISA with and without anti-IL-17A antibody represents the contribution of the bioactive fraction of IL-17A (9). IL-17A inflammatory and pro-coagulant effects have been well characterized on these HUVEC (5).

### Statistical Analysis

First, RA patients were divided into two groups according to their CVE status. Fisher’s exact tests were used to compare qualitative variables between these two groups. Mann-Whitney tests were performed to compare quantitative variables and bioactive IL-17A levels between CVE + and CVE- patients. A similar analysis was performed between destructive and non-destructive RA patients. Then, patients were divided into four groups according to their CVE status and wrist joint destruction score at sample collection and a Kruskal-Wallis test was used. Differences with *p*-values <0.05 were considered statistically significant. Statistical analysis were performed using GraphPad Prism version 8.4.3 (GraphPad Software, La Jolla, CA, United States)^1^.

## Results

### Patient Characteristics

Among 36 patients, 27 were females (75.00%), the mean age at sample collection was 61.89 (±SD 11.66) years, the mean DAS-28 at date of collection was 3.67 (±1.25), 58% of patients (19/33) were ACPA+, the mean wrist Larsen score at sample collection was 2.25 (±1.46). Twenty-three patients (64%) were considered as having a destructive RA (Larsen score ≥2). These patients were then followed-up for a mean duration of 19.81 (±11.55) years. The occurrence of a CVE, defined as myocardial infarction, stroke and peripheral acute ischemia, during follow-up was reported. Patients with a CVE (16/36 patients) had a large majority of myocardial infarction (9/16), followed by stroke (4/16) and peripheral acute ischemia (3/16). At sample collection, patients with CVE during follow-up were comparable with patients without CVE in terms of age, sex, ACPA positivity and DAS-28 (Table 1). Other patient characteristics are described in Table 1.

**TABLE 1:** Population characteristics.

**Population description (*N* = 36 patients)**					
**Variable**	**Values**			***p*-values**	**Missing data**
**Global characteristics**	**All patients (*N* = 36)**	**CVE+ (*N* = 16)**	**CVE– (*N* = 20)**		
Female sex, no. (%)	27 (75.00%)	11 (68.75%)	16 (80.00%)	0.47	–
BMI, kg/m^2^	26.16 ± 6.15	26.23 ± 6.73	26.10 ± 5.85	0.82	2 (5.56%)
Age at sample collection, years	61.89 ± 11.66	62.31 ± 10.44	61.55 ± 12.80	0.78	–
Length of follow-up, years	19.81 ± 11.55	22.69 ± 11.34	17.50 ± 11.47	0.19	–
CVE, no. (%)	16 (44.44%)	–	–	–	–
Myocardial infarction	9 (56.25%)	–	–	–	–
Stroke	4 (25.00%)	–	–	–	–
Peripheral acute ischemia	3 (18.75%)	–	–	–	–
RA characteristics at sample collection					
ACPA positivity, no. (%)	19 (57.58%)	10 (71.43%)	9 (47.37%)	0.29	3 (8.33%)
Wrist Larsen score	2.25 ± 1.46	2.88 ± 1.15	1.75 ± 1.52	0.015	–
Larsen score <2, no. (%)	13 (36.11%)	3 (18.75%)	10 (50.00%)		–
Larsen score ≥2, no. (%)	23 (63.89%)	13 (81.25%)	10 (50.00%)		–
DAS-28	3.67 ± 1.25	3.64 ± 1.31	3.69 ± 1.25	0.98	–
CV risk factors					
Never smoked, no. (%)	18 (51.43%)	6 (37.50%)	12 (63.16%)	0.18	1 (2.78%)
High blood pressure, no. (%)	21 (61.76%)	12 (80.00%)	9 (47.37%)	0.079	2 (5.56%)
Diabetes, no. (%)	6 (18.18%)	3 (21.43%)	3 (15.79%)	>0.99	3 (8.33%)
Dyslipidaemia, no. (%)	18 (60.00%)	10 (71.43%)	8 (50.00%)	0.28	6 (16.67%)
Treatments					
NSAIDs, no. (%)	23 (74.19%)	12 (92.31%)	11 (61.11%)	0.095	5 (13.89%)
Steroids, no. (%)	24 (68.57%)	13 (81.25%)	11 (57.89%)	0.17	1 (2.78%)
Methotrexate, no. (%)	33 (94.29%)	16 (100.00%)	17 (89.47%)	0.49	1 (2.78%)
Biologics, no. (%)	17 (48.57%)	8 (50.00%)	9 (47.37%)	>0.99	1 (2.78%)
Immunosuppressive agents, no. (%)	5 (14.29%)	4 (25.00%)	1 (5.26%)	0.16	1 (2.78%)
Other DMARDs, no. (%)	19 (54.29%)	7 (43.75%)	12 (63.16%)	0.32	1 (2.78%)

*Quantitative variables are reported as mean ± standard deviation. Fisher’s exact tests were used to compare qualitative variables. Mann-Whitney tests were used to compare quantitative variables. RA, rheumatoid arthritis; CVE, cardiovascular event(s); BMI, body mass index; ACPA, anti-citrullinated protein antibodies; DAS-28, disease activity score-28; NSAIDs, non-steroidal anti-inflammatory drugs; DMARDs, disease-modifying anti-rheumatic drugs.*

### Bioactive IL-17A Is Associated With CVE Occurrence and Destruction in RA

**FIGURE 1:**
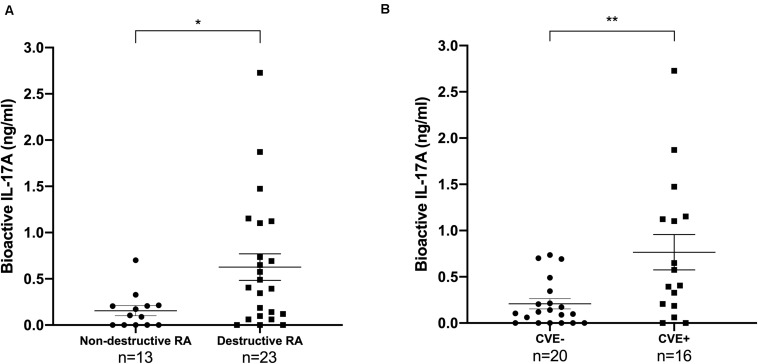
Bioactive IL-17A is associated with destruction and with the occurrence of CVE in RA patients. Thirty-six patients were tested for IL-17A bioactivity, reported as mean value (±SEM). **(A)** Patients were divided according to RA destruction [13 patients had non-destructive RA (Larsen score <2), 23 had destructive RA (Larsen score ≥2)]. Mann-Whitney test was used, *p < 0.05. RA, rheumatoid arthritis; IL, interleukin. **(B)** Patients were divided according to their CVE status (20 CVE- and 16 CVE+). Mann-Whitney test was used, **p < 0.01. CVE, cardiovascular event; IL, interleukin.

First, we confirmed that patients with destruction had higher bioactive IL-17A level than those without [0.63 ng/ml (±SEM 0.14) vs 0.16 ng/ml (±0.055), *p*-value = 0.018, Mann-Whitney test] (Figure 1A) (9). Bioactive IL-17A was higher in patients with CVE compared to those without [mean 0.77 ng/ml (±SEM 0.19) vs 0.21 ng/ml (±0.056), *p*-value = 0.0095, Mann-Whitney test] (Figure 1B).

**FIGURE 2:**
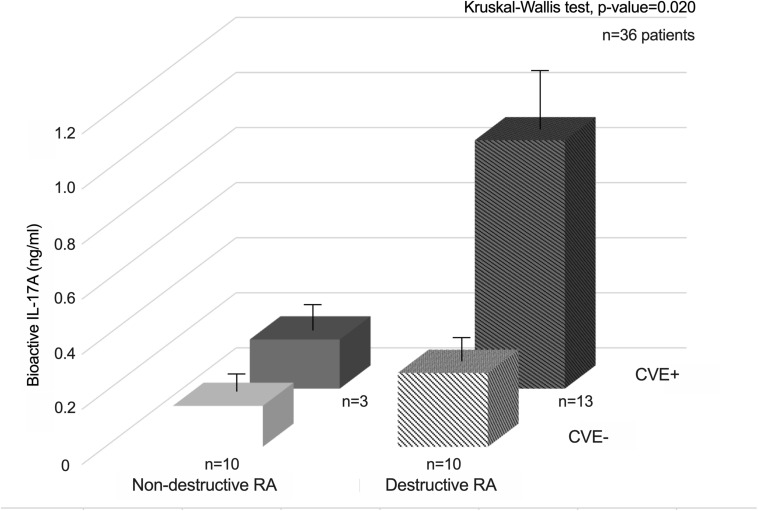
Synergistic interactions between bioactive IL-17A and joint destruction for the occurrence of CVE in RA patients.

Then, patients were divided into four groups according to their CVE status (16 CVE + and 20 CVE-) and wrist joint destruction score at sample collection (non-destructive RA with Larsen score <2, *n* = 13, and destructive RA with Larsen score ≥2, *n* = 23). Patients with both joint destruction and CVE had the highest bioactive IL-17A levels (*p*-value = 0.020, Kruskal-Wallis test, Figure 2).

## Discussion

These results showed for the first time that bioactive IL-17A was increased in RA patients who later experienced a CVE compared to those who did not. Results also suggested a synergistic interaction between joint destruction and CVE occurrence, through a common pathway associated with high IL-17A levels. It further demonstrates the dynamic continuum between local and systemic inflammation, leading first to destruction, and to increased CVE at later time points (4, 5, 9).

The role of IL-17A in RA pathogenesis was first evidenced from observations made on synovial tissue and fluid from RA patients (12). Then, *in vitro* and *in vivo* experiments confirmed that IL-17A mediates, synergistically with TNFα, cartilage destruction and bone erosion that characterized RA synovitis (13–15). Beyond its local detection into the synovium, bioactive IL-17A circulating level was also correlated with joint destruction in RA patients (9). This is particularly relevant given the fact that IL-17A has pleiotropic and systemic effects, especially on the CV system (16). Indeed, IL-17A, and moreover when combined with TNFα, induces endothelial dysfunction, vascular inflammation and atherosclerosis and finally promotes occurrence of CVE (5–7, 17). In a more acute situation, a peak of bioactive IL-17A occurred at the time of admission in patients with myocardial infarction (10). Therefore, the long-term release of IL-17A and TNFα from RA synovitis into the circulation can lead to systemic effects, especially on the CV system. This uncontrolled local inflammation might then contribute to the increased CV mortality observed in RA patients (16). While our study was focused on IL-17A specific role, other molecules are involved in RA pathogenesis and interact in a complex network. For instance, the role of TNFα, IL-6 and IL-1 is now well described in RA but also in the development of CV diseases (18). Results indicated that IL-17A amplifies the effects of TNF, IL-1, and others on matrix metalloproteinases, IL-6 and IL-8 production often in a synergistic pattern (19, 20). In this context, a cell-based bioassay was developed to emphasize the specific role of IL-17A in RA (9, 12).

Despite the relative low number of biological samples available, the long-term follow-up of the study is unusually high with the chance of having samples from entry. This allowed to draw a preliminary conclusion on the interaction between IL-17A, destruction and the CV risk. The study of CVE occurrence in RA is particularly complex because of the long-term follow-up that is needed, care changes over-time, loss of follow-up and heterogeneity of drugs. The benefit of IL-17 inhibitors was still unclear and these results illustrate the high disease heterogeneity regarding the possible contribution of this cytokine to the local and systemic expression of severe RA (8). Explanations partially came from many mediators that interfere with IL-17A function; some have synergistic effects (e.g., TNFα) and others antagonist ones (e.g., IL-25) (8, 16). For instance, the inhibition of IL-17RA, one of the subunit of the IL-17 receptor, also inhibits the anti-inflammatory effect mediated by IL-25, whose receptor shares one common subunit, IL-17RC, with IL-17A (4, 21). Moreover, in RA patients that do not achieve a suitable response to anti-TNFs, increased levels of IL-17 production and T-helper (Th) 17 cell frequencies were observed (22, 23). However, blocking IL-17A alone with secukinumab (an anti-IL-17A) after insufficient response or intolerance to anti-TNF was heterogenous but limited for the whole RA population (24–26). Using ixekizumab (another anti-IL17A), results were more promising in a phase 2 study and need to be confirmed (27). Given the synergistic interactions between IL-17A and TNFα, recent trials studied the dual inhibition of both IL-17 and TNFα pathways in RA patients with inadequate response to anti-TNFs (28). The IL-17 pathway was targeted in a more extensive way, using bimekizumab, an antibody that neutralizes both IL-17A and IL-17F (29). Certolizumab pegol was used to target TNFα either alone or combined with bimekizumab. The combination of both biologics allowed a better response in RA patients with an inadequate response to certolizumab pegol alone (28). These findings support that blocking multiple pathways may provide additional benefits in RA. In our context, the long-term evaluation of CV safety would be of a great interest.

Blocking directly pro-inflammatory cytokines with biologics is a way to lower inflammation but the use of other drugs, as statins, could be of a major interest in these patients exposed to chronic inflammation that developed premature CVE (30). For instance, statins acting through the cholesterol pathway were shown to inhibit the effects of IL-17 and TNFα on coagulation and platelet aggregation in cultured endothelial cells (31). Use of these classical and novel treatments could be another way to prevent the effects of IL-17 on blood vessels in these high CV risk RA patients.

Therefore, IL-17A, possibly of joint origin, may be one of the determinants of the CV risk in RA patients. The long-term follow-up over 15 years revealed that CV risk is determined at an early stage as shown by the predictive value of IL-17A bioactivity that synergizes with destruction. To confirm these promising results, the IL-17 story needs to be performed in larger and planned clinical trials and also in other chronic inflammatory diseases (8, 32). Its use may offer new perspectives in the prevention of CVE in inflammatory diseases and in the general population. One obvious limitation is the complexity of such long term follow-up.

## Data Availability Statement

The raw data supporting the conclusions of this article will be made available by the authors, without undue reservation.

## Ethics Statement

The study complied with the local Ethical Committee of the Hospitals of Lyon and was approved by the Ministry of Research (reference number: AC-2010-1164). The patients provided their written informed consent to participate in this study.

## Author Contributions

AH, MR, and NN-T were responsible for collection and assembly of the data. PM, AH, MR, FM, and NN-T undertook data analysis, interpretation, and critical revision of the manuscript. PM and AH were responsible for study concept and design. FM and MR performed the statistical analysis. PM and NN-T managed administrative, technical, and logistic supports. All authors contributed to the article and approved the submitted version.

## Conflict of Interest

NN-T and PM hold a patent on the determination of bioactive IL-17. The remaining authors declare that the research was conducted in the absence of any commercial or financial relationships that could be construed as a potential conflict of interest.

## Footnotes

1 www.graphpad.com
